# Recent Developments in Photoreceptor Optoretinography and Progress Toward Clinical Use

**DOI:** 10.1167/tvst.14.12.18

**Published:** 2025-12-15

**Authors:** Siyu Chen, David Huang, Ravi S. Jonnal

**Affiliations:** 1 Center for Ophthalmic Optics and Lasers (COOL), Oregon Health Sciences University, Portland, OR, USA; 2 Center for Human Ophthalmic Imaging Research (CHOIR), University of California, Davis Eye Center, Sacramento, CA, USA

**Keywords:** optical coherence tomography, adaptive optics, optoretinography

## Abstract

Optoretinography is an emerging class of methods designed to probe the function of retinal neurons using noninvasive, optical techniques. Early efforts sought to identify sources of functional signal and measure responses using advanced, research-grade imaging systems. In the past few years, some investigators have demonstrated simplified methods for measuring these signals using equipment very similar to what is used in commercial, clinical systems. The goal of this review is to introduce the reader to the sources of the photoreceptor optoretinographic signal and the technologies that have been developed to measure it.

## Introduction and Scope

Healthy, functioning photoreceptors deform in response to light stimuli. The magnitude of changes may be very low—the smallest reported responses are on the order of 10 or fewer nanometers—much smaller even than the length of a light wave. Accompanying this deformation may be changes in the reflectivity of the photoreceptor’s components. *Optoretinography* is the name of a growing class of methods devoted to measuring these subtle stimulus-evoked changes in the photoreceptors and other retinal neurons. Today, in the near absence of treatments for photoreceptor disease, the clinical significance of the optoretinogram (ORG) has yet to be established. However, novel therapeutics—including pharmaceutical, gene, and stem cell treatments—are being actively developed, and the ORG has the potential to streamline these efforts and eventual clinical trials of their products. Separately, the ORG is likely to find many applications in basic scientific research into photoreceptors.^1^ In this article, we provide a review of ORG methods developed over the past two decades. The intended audience is researchers and clinicians interested in photoreceptor function and dysfunction, and the goal of the review is to provide an overview of the existing technologies and a sense of how they may find their ways into commercial imaging systems or new research settings. The meaning of the term *optoretinography* is new and changing, so we must further specify the scope of this review.

We will limit this review to methods intended to observe the dynamics of the photoreceptor’s response to light stimuli. These dynamics may manifest in the reflectivity and/or movement of subcellular components of the photoreceptor. Necessarily, this limitation of scope excludes a lot of excellent work. Specifically, we do not cover changes in retinal reflectance that are solely attributed to photopigment bleaching (e.g., densitometry),^2^^–^^5^ and we do not cover much slower (>10 seconds) changes due to light and dark adaptation.^6^^–^^8^ To measure the dynamics of the photoreceptors’ responses, it is necessary to image them continuously while delivering a single stimulus flash^9^ or multiple flashes.^10^

We will also emphasize translational methods (i.e., those that could be used in patients). Some important work on animal models will be described in the next section, only because it represents early efforts to identify optoretinographic signals using methods suitable for clinical use. A more detailed review of foundational work conducted between the 1960s and 1980s in dissected animal retinae and suspensions of photoreceptors can be found in Jonnal.^11^

Finally, we must remind the reader that the results described below are not presented in chronological order. The section “Methods for Near-Term Clinical Translation” includes several methods that were developed more recently than the “advanced” ORG methods described later in the review.

## Anatomical Terminology

To understand the origins of the ORG signal requires differentiating the (sub)cellular components involved. Optical coherence tomography (OCT) provides an ideal in vivo tool that generates depth-resolved, cross-sectional images of the retina; therefore, measurements of scattering or morphological changes are attributable to specific retinal laminae. Generally speaking, in the macula, there are four outer retinal layers, which we will refer to as the external limiting membrane (ELM), inner–outer segment junction (ISOS; alternatively labeled the ellipsoid zone, EZ), cone outer segment tips (COSTs; also known as the interdigitation zone, IZ), and retinal pigmented epithelium (RPE). Although the attributions of the second and third layers to ISOS and COST have been contested,^12^^–^^16^ we use those terms here. Outside approximately 7° to 8° eccentricity, a fifth layer has been observed between the nominal COST and RPE layers, attributed to the rod outer segment tips (ROSTs),^17^^,^^18^ although with the benefit of cellular resolution through adaptive optics (AO), ROSTs can be visualized closer to the fovea.^19^^,^^20^

## Identification of Potential Optoretinographic Signals

The ORG signals described in this review consist of stimulus-evoked changes in either the light-scattering properties or morphology of the photoreceptor (or its subcellular components). An overview of the first evidence of these signals is given in this section. It should be said that the origins of the observed signal can be ambiguous. For example, apparent changes in the length of the outer segment (OS) could be due either to physical shrinkage/elongation or modulation of the refractive index, although changes in physical length have been reported using x-ray diffraction^21^ and microscopy.^22^ Moreover, changes in the apparent scattering of a retinal feature could be due to its movement away from the point of interrogation (e.g., a displacement of the hyper- and hyporeflective bands). It should also be said that both of these sources of signal may manifest in some light-stimulated structures. Despite these nuances, the two sources of signal are delineated and summarized here according to initial attribution.

### Stimulus-Evoked Changes in Scattering

Early OCT experiments revealed stimulus-evoked changes in the near-infrared (NIR) axial scattering profile of the retina in animal models. In dissected frog retina, Yao et al.^23^ identified a light-evoked reduction in scattering near the photoreceptors, but the specific photoreceptor layer was not reported. In dissected, rod-dominant rabbit retina, Bizheva et al.^24^ reported increases in scattering up to 80% from the distal photoreceptor region (possibly ROST and/or RPE) in response to a brief stimulus flash. The onset of the change was rapid (≪1 second) and the change persisted for 8 seconds.^24^ In anesthetized living rats, Srinivasan et al.^25^ reported a 12% increase in scattering centered between the ISOS and the ROST/RPE complex, appearing to span the length of the outer segment. The corresponding OCT intensity rose sharply in the initial 0.5 seconds, and kept increasing over the duration of measurement (∼4 seconds). In mice, Zhang et al.^26^ observed increases in scattering from all photoreceptor layers, in response to a brief stimulus flash that bleached between 0.04% and 95% of rod photopigment. The reported increases in ISOS and ROST were significantly higher than previous studies, with ISOS scattering increasing by a factor of 5 to 10 and ROST increasing by a factor of 3, both changes taking place over approximately 100 seconds. In living leopard frogs, Zhang et al.^27^ identified increases and decreases in the OCT amplitude in single pixels in the cone and rod OS layer, in response to a brief 10-ms, 532-nm flash. These changes persisted for hundreds of milliseconds and had relatively large amplitudes—exceeding ±100% of the baseline amplitude—which is consistent with speckle modulation due to subresolution rearrangement of scatterers. Their finding illustrates the possibility that morphological changes contribute to observed changes in OCT amplitude. The inconsistency among these results could be partly explained by methodological differences. Bizheva et al.^24^ reported changes throughout the retina, which led them to highlight the changes with the largest absolute magnitude. Srinivasan et al.,^25^ on the other hand, focused on scattering changes centered, axially, on the relatively dim OS band. Comparisons are further complicated by the speculative attributions of image features to anatomical structures, and there may have been differences in attribution among research groups. Other factors not considered at that time were the extended axial point spread functions (PSFs) of the systems and potential mixing of axially distinct signals, differences between in vivo and ex vivo responses, and, as mentioned above, the possibility that a layer's movement was mistaken for a scattering change. By the time of Zhang et al.,^26^ many of the questions of attribution had been resolved, and a methodology for tracking movement and scattering simultaneously had been devised. The changes reported in that paper could, in principle, have underlaid the changes reported in Bizheva et al.^24^ and Srinivasan et al.^25^

### Stimulus-Evoked Changes in Morphology

Although not discussed in their publication, the M-scans (i.e., repeated A-scans at the same transverse imaging location; analogous to M-mode ultrasound for motion detection) published by Bizheva et al.^24^ are consistent with morphological changes near the ROST or RPE (their fig. 1d).^24^ Soon after, Jonnal et al.^9^ showed that a visible stimulus flash changed the coherent reflectance of the cone OS, implying that its length was affected by stimulus and that this response initiated within a few milliseconds of the flash. Zhang et al.^26^ showed that in response to a brief visible flash, mouse rods exhibit an increase in the separation between the ISOS and a deeper layer (with primarily contribution from ROST and RPE). They attributed this change to OS elongation. This elongation had a magnitude of approximately 2 µm, occurred over approximately 100 seconds, and persisted for the 300-second duration of measurement (their figs. 1D, F).^26^ In a later work, they modeled the mixed ROST/RPE reflection with a Gaussian mixture model and confirmed that the ISOS-ROST distance increased by 2 µm after light stimuli, although they also noted some diurnal variation in ORG responses.^28^ Stimulus-evoked changes in both OS length and subretinal space (SRS) length were observed in a rat model by Tan et al.,^29^ using phase-sensitive OCT. In their OCT images of the rat retina, a single band was hypothesized to contain contributions from ROST and RPE. By monitoring phase differences between pixels in that band and the ISOS, a machine learning algorithm was able to classify the pixels into two categories of response, one exhibiting elongation within the first second, followed by contraction, and another exhibiting elongation over 10 seconds. These were hypothesized to originate from the rod OS and SRS, respectively. Together, these works suggested that light stimuli caused movement of reflective structures in and around the OS and revealed a possible new biomarker of photoreceptor function.

## Technical Specifications Affecting Clinical Translatability

This review aims to classify proposed optoretinographic methods by their suitability for clinical translation. While there is no single standard for clinical translatability, in this review, we will consider whether (1) the cost of the equipment is comparable to what is currently spent by clinics on imaging devices, (2) the equipment can be operated by a single trained ophthalmic technician, and (3) the macula can be tested and the results of testing can be provided to the clinician during the average 20-minute^30^ patient visit.

Broadly, this divides the currently available ORG approaches into two categories: category I (those with the speed and resolution of clinical ophthalmoscopy, measuring macroscopic changes in tissue) and category II (those requiring significantly higher speed, the resolution of single photoreceptors, or both). Cellular resolution depends on optical aberration correction, either with a hardware wavefront modulator or using digital methods. Category II instruments generally have a limited field of view (1–2°), to accommodate the high sampling density required for cellular measurements and to avoid loss of resolution through anisoplanatism.^31^ While surveys of the macula are possible, measurement durations are long, and the resulting volumes of data require hours of postprocessing. By contrast, category I methods have been demonstrated using commercially available instruments (or prototype instruments with comparable performance envelope) with minimal modifications or prototype instruments that fulfill the criteria listed above.

In the next sections, we will first describe demonstrations of clinically translatable approaches and then describe advanced approaches that are currently better suited to basic scientific inquiry and small sample studies. They are not described in chronological order.

## Methods for Near-Term Clinical Translation

In this section, we describe a few ORG methods utilizing OCT hardware comparable to what is available in commercial ophthalmic imaging systems. Specifically, we highlight findings achieved with fundus cameras and scanning OCT systems, without AO or digital aberration correction (DAC), and imaging speeds comparable to commercial instruments.

### Methods Measuring Reflection/Back-Scattering Changes

Light-evoked changes in photoreceptor back-scattering can be observed using most ophthalmic imaging techniques, including fundus cameras and OCT. Here we will limit our discussion to optical signals occurring within a few seconds, as these should be most related to the processes of pigment photoisomerization and subsequent activation of the phototransduction cascade.

The expected change in image intensity is small with respect to the steady-state reflectivity. Therefore, researchers often detect changes in pairs of measurements—one set obtained at the baseline (or at an unexposed retinal area) and the other after the presentation of light stimuli. In 2006, Abramoff et al.^32^ used a modified fundus camera to measure retinal reflectance change in the NIR region (780 nm). The visual stimulus measured approximately 10 cd/m^2^ and was presented in a randomized 3-second-off/3-second-on sequence. Results were averaged over five stimulus cycles. A time-dependent decrease of 0.1% to 0.3% was observed following the onset of stimulus, where the peak change occurred approximately 1.5 seconds after exposure to light and showed a gradual return to the baseline at the end of the 6-second cycle. The tested region spanned 45° of visual angle and thus consisted predominantly of peripheral retina. It should be noted that the stimulus level was significantly lower than stimuli used in later experiments.

Srinivasan et al.^33^ reported the first depth-resolved measurements of stimulus-evoked scattering changes in human volunteers. They used a prototype OCT instrument with approximately 3.3 µm axial resolution and measured changes in the reflectance of the ISOS and ROST in response to a 1-second stimulus of 5 × 10^4^ Td. They reported a statistically significant, gradual 2% to 3% increase in ISOS scattering during light stimulation in parafoveal and perifoveal retina, and persisting for the 1.5-second duration of measurement. They did not observe this increase in the peripheral retina. In the perifovea and peripheral retina, 3% to 5% reductions in ROST scattering were observed in response to a 0.5-second stimulus. These changes occurred over approximately 0.5 seconds and persisted for several seconds. Averaging was performed across multiple trials (up to nine) and, for investigating the temporal dynamics, across the entire simulated field.

A similar strategy was employed by Schmoll et al.^10^ Instead of repeating individual trials, they used a flickering stimulus at 5 Hz and bandpass-filtered the scans in the temporal dimension. By isolating OCT changes that fell within the same frequency range as the stimulus, they observed a 2% to 3% increase in back-scattering from both the ISOS and COST.^10^ Their measurements were taken between 3° and 5° from the foveal center.

Son et al.^34^ observed increases in the OCT amplitude at the ISOS and at the OS/RPE interface in response to a visible white flash. These responses were fast, occurring within milliseconds of the flash, and lasted for hundreds of milliseconds. The flash duration and illuminance were not reported. In closely related work, Ma et al.^35^ visualized separately the amplitude and phase components of stimulus-evoked dynamics in the retina, using serial B-scanning at 100 or 1000 Hz.^35^ The stimulus flash duration was not reported. The amplitude changes were quantified by subtracting the prestimulus B-scan from the poststimulus B-scan and integrating the absolute difference from the ELM to the RPE. Phase changes were computed similarly, by unwrapping the OCT phase axially, subtracting a constant phase ramp, and integrating the absolute difference over the same region.

A frequent observation in human and animal studies has been an increased OCT amplitude of the band attributed to the ISOS layer in response to visible stimuli. However, measurements of other layers (OS, COST, ROST, and RPE) have been somewhat inconsistent, with possible dependencies on eccentricity, photoreceptor type, and species.

Some investigators have adopted less specific, but perhaps more robust, probes of light-evoked changes in scattering. Instead of quantifying amplitude changes in specific, labeled outer retinal layers, they developed ways of aggregating light-evoked changes in scattering of any sort. This kind of method–one that is partly or entirely agnostic about the structural integrity of the outer retina–may prove to be especially useful in diseases that disrupt the outer retinal structure and preclude segmentation of the typical ORG layers of interest.

Specifically, Chen et al. employed a method analogous to split-spectrum amplitude-decorrelation angiography (SSADA)^36^ to quantify stimulus-evoked changes in the image by calculating the decorrelation of the OCT amplitude after light exposure, terming it split-spectrum amplitude-decorrelation optoretinography (SSADOR).^37^ This method is motivated by the fact that even relatively small changes in scatterer position and reflectance can lead to substantial alterations in the OCT speckle pattern due to coherent summation. Similar to earlier SSADA studies, tuning the effective OCT bandwidth (i.e., the axial resolution) improves suppression of noise caused by bulk eye movements, while allowing isolation of signals from a specific OCT band (i.e., the cone outer segment). In this work, the authors reported a ∼30% increase in decorrelation signal and 25% improvement in ORG SNR with a four-way spectrum split. Their study used a prototype SD-OCT operating at a 250-kHz A-scan rate, which repeatedly scanned a 10° × 3.3° field of view (FOV) at 2 Hz. A flash with vertical black-and-white stripe pattern, lasting 200 ms, bleached approximately 15% of exposed cone pigments at the beginning of the third OCT volume. Peak decorrelation was observed approximately 0.5 seconds following the exposure and lasted beyond the 1.5-second imaging period.

### Methods Measuring Optical Path Length Changes

OCT is a phase-sensitive imaging technique (i.e., it records the optical phase of light back-scattered from the sample, which is highly sensitive to movements of the sample). When the sample is stabilized and movements are known to be small, the OCT phase can be used to quantify them.^38^ However in retinal OCT imaging, the phase information is often discarded, for two reasons: (1) phase changes due to microscopic movement of cellular structures are swamped by those due to movements of the head and entire eyeball, and (2) serial phase measurements from a single retinal feature suffer from a 2π ambiguity due to the cyclic nature of the optical phase. The latter problem can be addressed by utilizing an imaging speed sufficient to guarantee that movements are smaller than about half the imaging wavelength. This approach was previously demonstrated in Doppler OCT, which infers blood velocity from such phase shifts.^39^ Even when unquantifiable, ambiguous phase changes can serve as a source of contrast, as in phase-variance OCT angiography (OCTA).^40^ In these cases, however, the former problem remains. In phase-variance (and complex variance) OCTA and Doppler OCT, the bulk motion of the retina (due, for instance, to an axial head movement) can be estimated and numerically corrected using statistical methods, because it usually presents as a slow trend over the scanned width of a B-scan.^41^^,^^42^ Alternatively, bulk motion can be factored out by monitoring relative phase differences among structures on the optical axis rather than their absolute phase.^43^ Specifically, to measure light-evoked changes in the length of the OS, the phase of the ISOS is subtracted from the phase of the outer segment tips (COST in cones and ROST in rods). The latter approach has been used in all of the phase-based photoreceptor ORG methods.

Advanced ORG methods (described later) were originally employed to quantify the magnitude and speed of light-evoked deformations of the cone and rod OS. Resulting measurements^44^^–^^46^ suggested that imaging rates above 100 Hz (i.e., below a 10-ms temporal resolution) would be required to measure the early contraction stage of the response, which lasts approximately 20 ms. For a commercial OCT system (or comparable research prototype) generating A-scans at rates around 100 kHz, serial volumetric scanning would permit volumes of only 1000 A-scans each, which would severely compromise either sampling density or field of view. Serial B-scans, by contrast, would permit adequate sampling density over larger fields of view, but phase changes over hundreds of milliseconds would be dominated by the combined effect of lateral eye movements and the natural topographic variation in the retinal layers of interest.

Vienola et al.^47^ demonstrated a novel ORG method based on serial B-scans. They assumed that the retina is effectively stationary over sufficiently small time spans and divided the entire series of B-scans into blocks each acquired within 7.5 to 12.5 ms. Within those blocks, instead of monitoring the relative phase differences (i.e., positions) of the ISOS and COST layers, they computed their rate of phase change (i.e., velocities). Under the assumption that those velocities would be constant over the stimulated (1.2°) region, this method can tolerate eye movements occurring over the entire series, provided they are smaller than the stimulated region. Their results confirmed what had been shown with advanced systems, that the cone OS contracts over the first ∼20 ms after stimulation and elongates after that. They showed that these components of the response increase with increasing stimulus dose and decrease with increasing retinal eccentricity. As expected, the integral of the relative velocity resembles the position-based measurements described below. The method appears to be highly translatable given its low cost and complexity. However, the assumption of motionlessness within small intervals is fundamentally false, and eye movements normal to the B-scan plane represent an uncorrectable source of phase noise.

## Advanced Methods for Basic Scientific Inquiry and Future Translation

To date, the most sophisticated ORG implementations are capable of resolving single cells and measuring their responses to stimuli individually or in aggregate, with high temporal bandwidth. These methods have been propelled by technical improvements in speed and/or resolution. We will start with an overview of the technical challenges and advancements and then describe how these have been used in ORG investigations of the photoreceptor response and its hypothetical origins.

The more prominent approach uses AO to correct high-order optical aberrations of the eye, thereby allowing near-diffraction-limited imaging resolution of 2 to 3 µm (imaging in the 700-nm to 1-µm band with a 6.5-mm to 7.5-mm dilated pupil).

Measurements in single cells offer unparalleled scientific potential. Even when averaged to reduce noise, these measurements offer advantages over low-resolution methods that perform such aggregation optically, since the latter are more susceptible to ambiguity in axial and lateral localization of signal. Notwithstanding their added complexity and cost, their ability to measure function in single cells may confer a translational advantage too, since this is where disease first manifests.

### The Spatial Scale of Photoreceptors

The spacing of cone photoreceptors ranges from 2 to 10 µm, and that of rods is even smaller (approximately 1 to 4 µm outside the rod free zone).^48^ Cones in the foveola, responsible for high-acuity vision, are no more than 3 µm apart. Foveal cones and rods cannot be resolved with conventional imaging systems, regardless of the size of the imaging beam. Small beams result in diffraction-limited resolutions larger than the cells, and large beams passing through a dilated pupil suffer the effects of ocular aberrations and result in large, time-varying point-spread functions. Visualization of foveal cones and rods requires dilation of the pupil and imaging with a large beam, while simultaneously compensating for the effects of ocular aberrations. Initially, aberrations were compensated with real-time adaptive optics subsystems,^49^^,^^50^ which allowed visualization of many previously unresolvable structures^51^^–^^54^ and countless studies of their structure in health and disease.^55^ More recently, it was shown that digital methods can be used to correct aberrations in images that contain information about the phase of the light scattered from samples, such as holograms^56^ and OCT images.^57^^–^^59^ The latter is especially true in the case of full-field OCT, where phase shifts due to axial movement of the sample do not confound the estimation of aberrations.^44^

### The Temporal Dynamics of the Photoreceptor Response

Early work had shown that observable changes in photoreceptor reflectance (hypothetically attributed to OS length changes) occurred within a few milliseconds of stimulus,^9^ and much was already known about the high temporal bandwidth of cones, which has been measured to be 60 Hz using psychophysical tools and up to 90 Hz using single cone electrophysiology.^60^ Assuming that the morphological response of the cones was related to their function, OCT volume rates in the tens or hundreds of Hz would be required to quantify these cellular responses. Several measurements of stimulus-evoked OS elongation^45^^,^^61^^,^^62^ revealed elongation velocities on the order of hundreds of nanometers per second after bright stimulus flashes. To measure these velocities without suffering the 2π ambiguity described above, ORG sampling rates in the tens of Hz would be required. The revelation of an early, fast (∼20 ms) contraction of the cone OS^45^^,^^46^^,^^61^ demonstrated a need for even higher ORG sampling rates, 50 to 100 Hz or more. The majority of ORG studies have employed areal imaging (i.e., volumetric in the case of OCT and en face in the case of fundus cameras), but no existing clinical imaging system is capable of producing areal images at those rates.

Several areas of technical innovation have enabled the development of high-speed ORG modalities. In swept-source OCT, the A-scan acquisition rate is limited by the sweep rate of the laser. In the past 10 years, high-speed (>400 kHz) sources have emerged based on micro-electromechanical systems (MEMS)–based tunable filters,^63^ vertical-cavity surface-emitting lasers (VCSELs),^64^ and Fourier-domain mode-locked (FDML) lasers.^65^ Raster-scanning OCT and AO-OCT systems have been implemented using these fast sources and used to measure photoreceptor ORG responses. In spectrometer-based OCT, the A-scan acquisition rate is limited by the speed of the line-scan cameras used in the spectrometers. Kocaoglu et al.^66^ combined four 250-kHz spectrometers with an optical switch to create an OCT system with a 1-MHz A-scan rate. As of now, an advantage of high-speed spectrometers over high-speed swept-source lasers is the availability of broadband low-coherence sources (superluminescent diodes [SLDs]) in the 600- to 1000-nm band. High-speed swept sources suitable for retinal imaging are currently only available in the 1000- to 1100-nm wavelength range. Because lateral resolution is proportional to wavelength and axial resolution is proportional to its square, spectrometer-based methods at present offer higher lateral and axial resolutions.

In parallel with these innovations in scanning systems, there have been many developments in the area of full-field (FF) swept-source (SS) OCT. The concept was proven using stationary samples and a relatively slow (15 Hz) camera,^67^ but it was not until the introduction of sufficiently sensitive CMOS cameras with much faster frame rates (10–1000 kHz) that it was demonstrated in the living eye.^44^^,^^68^ In FF-SS-OCT, an extended region of the retina is illuminated, and the resulting image is interfered with a similar image of a reference mirror. If the light source is swept over a range of wavelengths while a high-speed camera acquires a spectral series of images, the latter can be used to reconstruct a three-dimensional (3D) volumetric image. Because all of the A-scans are collected in parallel, this approach achieves high volume rates (>100 Hz) and phase stability of the resulting volumes.

Two-dimensional cameras have also been used to demonstrate line-scanning spectrometer-based (LS-SD) OCT systems, in which one dimension of the sensor is used to encode spatial information and the other dimension spectral information.^69^^,^^70^ All A-scans within a B-scan are obtained simultaneously; however, the different B-scan locations are acquired sequentially using a scanning mechanism. Each resulting space-wavelength (x–λ) frame is converted into a B-scan, resulting in B-scan rates at the frame rate of the camera. The high-speed cameras available in the last 10 years have permitted B-scan rates in the tens of kilohertz.^71^

### ORG Implementations Utilizing High-Speed OCT Imaging

The speed of OCT systems is usually expressed as A-scan rate (for scanning systems) or effective A-scan rate (for parallel detection systems). Since its advent, the speed of ophthalmic OCT systems has increased from A-scan rates of 0.8 Hz^72^ to A-scan rates of 3.2 MHz^73^ and effective A-scan rates in full-field systems of tens of megahertz.^44^ Such improvements in speed have enhanced ORG measurements by supplying higher temporal bandwidths, sampling densities, and fields of view. Temporal bandwidth can be used to measure responses to strong stimuli capable of causing rapid phase changes or to study fast dynamics such as the initial OS contraction or responses to high-frequency flicker. Higher sampling density can improve phase sensitivity through averaging. Larger fields of view, in principle, improve the clinical translatability of the methods. Jiang et al.^74^ employed a line-scanning approach without AO to demonstrate high-speed (16-kHz B-scan rate, 32-Hz volume rate) ORG measurements over a moderate (5.5°) field of view. They were able to reliably measure the elongation stage of the cone response and to show that the maximum elongation of the OS fell with increasing retinal eccentricity. Importantly, they were able to establish this eccentricity-dependence from a single acquisition. While the early contraction stage of the cone response was visible in some of their plots, it was not reliably observed, as predicted by that stage’s brief (∼20 ms) duration.

Ni et al.^75^ advanced the SSADOR technique by employing a 1-MHz MEMS-VECSL laser. This permitted acquisition of volumes over a 10° × 10° FOV at a rate of 1.67 Hz, representing a ∼3× increase in the FOV area from their earlier report,^37^ with only a small reduction in the volume rate. Spatially varying stimuli (i.e., images) were delivered using a projector. The decorrelation of stimulated parts of the retina was significantly higher than unstimulated parts, and in trials where the stimulus intensity was varied spatially, decorrelation rose with increasing bleaching percentage. Decorrelation increased by ∼60% between bleaching levels of 0% and 26%.

Gong et al.^76^ used a 600-kHz MEMS-VCSEL swept-source to measure photoreceptor responses to flicker stimuli and adopted an analytical approach motivated by earlier FF-SS-OCT experiments.^77^^,^^78^ Optimizing the trade-offs between FOV and volume repetition rates, they scanned a 4° × 1.33° region at 10 Hz before flicker onset and a 4° × 0.27° region at 48 Hz to measure the responses during flicker stimulation. During the first stage, the retina was exposed to a constant amount of stimulus light, equal to the average illumination of the flicker. The two-stage approach caused subjects to reach a steady-state bleaching level prior to flicker measurement, which obviates the need for dark adaptation. The larger prestimulus volumes also provided the spatial reference for image registration. Photoreceptor responses were tracked using the OCT phase difference between the ISOS and COST layers. Using Fourier analysis, the authors isolated and quantified the response amplitude corresponding to the flicker frequency. In all subjects, response amplitude was shown to decline with increasing frequency. They also showed that response amplitude was higher at 4° eccentricity than that at 2°.^47^^,^^74^

### ORG Implementations Based on High-Resolution En Face Imaging

En face (two-dimensional [2D]) imaging modalities offer some inherent advantages over 3D counterparts. Notwithstanding limitations on speeds of resonant scanners traditionally employed in scanning light ophthalmoscopes (SLOs), 2D imaging modalities are in principle faster than 3D ones. By producing fewer data, they reduce demands for data processing and storage, which could enhance their translatability. Of course, they attain these advantages at the expense of depth resolution.

Jonnal et al.^9^ used high-speed (200 Hz) AO fundus imaging, which revealed flash-evoked changes in the reflectivity of cone photoreceptor mosaic. They observed an oscillating reflectivity change of the cone photoreceptors following a brief light flash under coherent illumination conditions. They hypothesized that the photoreceptor outer segment acted as a common path interferometer, where alternating constructive and destructive interference occurs as the segment elongates, creating changes in measured reflectivity.

AO fundus cameras have the potential to be fast, but they lack the ability to reject unwanted light. Light scattered from all layers of the retina, as well as multiply scattered light, comes back to the sensor, reducing the image SNR as compared to AO-SLO imaging.

Cooper et al.^79^ used an AO-SLO to demonstrate an effect similar to Jonnal et al.^9^ and devised a way to pool photoreceptor responses to generate a reproducible, quantifiable signal. Using this approach while varying the wavelength of the stimulus flash, they were able to measure the action spectra of the ORG responses, which they showed agreed with the standard observer luminosity function (V(λ)). They later demonstrated that reproducible signals from individual cells could be generated by measuring responses multiple times (number of averaged trials was 13 in the study) and pooling the responses from each cell, and they used this method to identify S-cones and validate that classification using densitometry.^80^ Other investigators have used this approach to demonstrate an eccentricity-dependence of the pooled cone response, with cones at greater distances from the fovea exhibiting a smaller response than those close to the fovea.^81^ While AO-SLO is better at rejecting stray light than AO fundus cameras, the technology is limited by the speed of the resonant scanners they use to scan the beam in the fast dimension. These have generally limited AO-SLO systems to speeds under 30 Hz, too slow to measure the fastest components of the ORG response.

Other investigators have leveraged speed to study light-evoked, incoherent changes in photoreceptor reflectance. Bedggood and Metha^82^ used an even faster (1 kHz) AO fundus camera to image cone reflectance changes in response to the visible (540–570 nm) illumination source.^82^ Their method permitted measurement of densitometric effects (i.e., overall increase in cone reflectivity) combined with higher-frequency changes in reflectance. Because they had measured the coherence length of the source to be 7 µm—shorter than the OSs they imaged—they hypothesized that these changes were not due to elongation of the photoreceptor but rather to other effects. However, the elongation hypothesis cannot be completely ruled out, because scatterers found within the OS^83^ may also contribute.

### ORG Implementations Using High-Speed and High-Resolution

Finally, we will cover the most sophisticated ORG implementations. These are the methods that have elucidated the photoreceptor ORG response in greatest spatiotemporal detail. The first measurements of what we now consider the “cone ORG response” were done using such systems. We will first give an overview of such systems and their capabilities, and then describe the scientific applications that they have facilitated.

#### Technical Characteristics of High-Speed, High-Resolution ORG Implementations

The first OCT systems capable of measuring single cells consisted of AO systems coupled to a raster scanning, linear spectrometer OCT system^84^ or a 2D (x–λ) spectrometer OCT system.^69^ Until relatively recently, the former approach predominated in the high-resolution OCT community. Initially, raster-scanning systems employed spectrometers and were thus limited by the speeds of their line-scan cameras. Kocaoglu et al.^66^ overcame the limit imposed by a single such camera by multiplexing four 250-kHz spectrometers using an optical switch and achieved an A-scan rate of 1 MHz. The advent of Fourier-domain mode-locked tunable sources combined with high-speed digitizers provided an alternative to spectrometer multiplexing,^65^ although these were not commercially available until the early 2010s and continue to be limited to center wavelengths around 1 µm. However, they were successfully combined with AO systems and used to acquire cellular-resolution images of the retina, achieving speeds of 1.6 MHz^85^ and 3.2 MHz.^86^ These implementations achieved volume rates between 6 and 30 Hz, sufficient for recording the stimulus-evoked elongation of the cone OS.^20^^,^^45^^,^^62^^,^^86^ The fast early contraction of the cone OS could not be observed at these volume rates but was revealed by examining serial B-scans of single cones acquired at 3 kHz.^45^

An alternative to scanned, serial A-scan acquisition is parallel detection using 2D sensors, where two forms of parallelism have been demonstrated: full-field, in which the dimensions of the sensor encode spatial information (x–y) and spectral information is encoded temporally, or line-scan, where the two dimensions of the sensor encode a spatial dimension and the spectral dimension (x–λ) while the other spatial dimension is encoded temporally (i.e., scanned).

Development of FF-SS-OCT represented a major improvement in the speed of high-resolution OCT systems. These systems have the advantage of spatial phase uniformity, since, unlike in raster scanning implementations, movement of the sample impacts the phase of all A-scans uniformly. This advantage, combined with OCT's ability to record both the amplitude and phase of the sample wave, permitted optical blur to be corrected using digital wave propagation methods (scalar diffraction methods such as Rayleigh or Fresnel integrals) and optimization of the pupil function (measured or propagated) phase.^44^^,^^87^ FF-SS-OCT has also been combined with real-time hardware AO,^88^ which reduces the computational demands in processing and possibly permits resolution of smaller cones closer to the fovea. The other advantage of parallel detection was a 10-fold increase in effective A-scan rates, which enabled the first OCT volume rates of more than 100 Hz.^44^ A disadvantage of FF-SS-OCT is that serial detection of the spectral dimension permits sample movement, and system vibrations result in chirp of the recorded OCT fringe and subsequent loss of contrast in the spatial domain, but numerical methods have been demonstrated—similar to those employed to correct mismatch of dispersion in the sample and reference arms.^89^

Parallel detection of the spectral dimension is achieved in line-scan OCT with 2D (x–λ) sensors. Initial efforts to achieve cellular resolution with this approach employed digital aberration correction,^90^ but this method was complicated by the serial detection of one spatial dimension and subsequent motion-induced phase instability of the OCT volume. Recently, the approach has been combined with hardware AO, achieving cellular resolution and an effective A-scan rate of 8 MHz.^71^ Both parallel detection schemes possess sufficient bandwidth to detect and measure the early contraction stage of the cone response.^44^^,^^46^

#### ORG Applications of High-Speed, High-Resolution OCT

Hillmann et al.^61^ used an FF-SS-OCT system in conjunction with DAC to reveal the fundamental features of the cone ORG. Their system acquired volumetric images of the retina at up to 167 Hz, in which they showed that the phase difference between the COST and ISOS changed after stimulation, corresponding to an initial contraction, followed by an elongation of hundreds of nanometers lasting hundreds of milliseconds. One limitation of their study was the lack of measurements taken within the fovea, due in part to the increased computational demand imposed by digital correction of high-order aberrations. Full-field OCT is also vulnerable to cross-talk noise, in which signal originating from a point in the retina is modulated by coherent interference from neighboring points within the lateral PSF of the system.

To address these limitations, a recent FF-SS-OCT development incorporates two important novel aspects. Tomczewski et al.^77^ devised a method for reducing the spatial coherence of the illumination source, using a long multimode fiber (300 m) to reduce the spatial coherence of the illumination source, effectively suppressing the contribution of multiply scattered light from areas further apart and therefore improving imaging SNR. A second key innovation of their work was the delivery of flicker stimuli rather than single flashes. In their implementation, they were able to image a FOV of 5.7° × 2.8° at a volume rate of nearly 200 Hz, while delivering flickering stimuli at rates between 15 and 30 Hz. They were able to show that Fourier transformation of the resulting COST-ISOS phase difference resulted in clear peaks at the flicker frequencies, and that the amplitude of the ORG response fell with increasing frequency. In a later work, they showed that by delivering flicker stimuli with time-varying frequency (i.e., chirp), they could use the same method to measure the frequency-dependence of the ORG response, which could serve as an independent biomarker of photoreceptor health.^78^ Most recently, they used characteristics of the flicker response in humans as well as reduction in the single-flash response in mice after treatment with phosphodiesterase-6 (PDE6) antagonist sildenafil to motivate a hypothesis that the stimulus-evoked elongation of the outer segment is partly due to (PDE6) activity. This is similar to the hypotheses advanced by Zhang et al.^26^ in mouse rods and Pandiyan et al. ^91^ in human cones, but differs in that it does not attribute the swelling entirely to the osmotic side effects of hydrolysis of guanosine triphosphate (GTP) in the G protein–phosphodiesterase complex, but rather a mechanical effect of PDE6’s conformational change on the interdisc spacing.^92^

The second recent FF-SS-OCT application is its combination with closed-loop, hardware AO, which was used to test several models of the ORG response to single flash stimuli and more complex stimuli.^88^ The most complex of the proposed models predicts three stages of the OS response: early contraction, elongation, and late contraction. Comparison of more and less complex models showed that the resulting time constants depend on the complexity of the model and, specifically, that failing to model the late contraction of the OS leads to inaccuracies in characterizing the earlier stages.

While raster-scanning OCT cannot achieve as fast speeds as parallel detection, they have several unique capabilities, (e.g., the scanning protocol can be easily adjusted to suit imaging needs). They are compatible with both swept-source and spectral-domain detection. They also tend to have the best SNR performance among all three configurations because the optical confocal gating efficiently rejects out-of-focus light. Azimipour et al.^62^ developed a 2D raster-scanning AO-OCT system based on a 1.6-MHz FDML laser, capable of scanning a ∼1° × 1° FOV at 32 volumes per second. They used this system to show that responses were visible in single-cone photoreceptors, using the COST and ISOS phase as they had previously proposed,^43^ and that these responses scaled with the stimulus dose. In addition, they showed that light stimuli cause movement of portions of the ISOS and RPE bands, visible in the amplitude of the OCT image of single cells. The use of hardware AO permitted measurements to be taken as close as 1.5° from the foveal center. Cones closer to the foveal center could not be resolved, due in part to the system’s 1-µm imaging wavelength.

Around the same time, Zhang et al.^45^ used a multiplexed spectrometer-based AO-OCT system that achieved A-scan rates of 1 MHz. They used this high A-scan rate to acquire volumes at 10 Hz over a 1° × 1° FOV. They too found a dose-dependent elongation of the OS. Their system scanned in the fast dimension at 3 kHz, and by comparing the ISOS and COST phase in adjacent scans of the same cone, they were able to study the early OS contraction with high temporal resolution. They reported a nonlinear dependence of elongation slope and amplitude on dose, as well as a linear dependence of contraction amplitude on dose. From the latter, they reasoned that the contractile stage was related to photopigment activation—a hypothesis that would later be echoed by other investigators.^46^^,^^91^ They observed a trimodal distribution of cone responses to stimuli of various colors (blue, green, and red). By concatenating cone responses in spectral order and subjecting the resulting response trains to principal component analysis, they were able to classify cones with uncertainty rates much lower than that of AO densitometry. They validated the classification method by showing that in a deuteranopic subject (lacking M-cone pigment), virtually none of the cones were classified as M-cones.

Later, Azimipour et al.^20^ modified their AO-OCT by reducing the speed of their fast-dimension scanner, thereby lowering their volume rate to 6 Hz while increasing their sampling density on the retina.^20^ In addition, they added an AO-SLO channel at 840 nm, which benefited from improved lateral resolution at that wavelength. The two systems acquired data in parallel. The AO-SLO images resolved cones and rods, while the AO-OCT system, with its 1-µm imaging wavelength, lacked the resolution to resolve rods. Nevertheless, using the SLO images as a guide, they were able to segment blurred regions of the OCT volume consisting of rods, and measure the responses of rods to stimulation. They found that, like cones, rods elongate after visible stimulation. Their 6-Hz volume rate precluded observation of an early contractile stage. As with cones, elongation of rods increased with increasing stimulus energy, with possible saturation after 4% rhodopsin bleaching. For similar bleaching percentages, the elongation velocity of rods was lower than that of cones while the amplitude of elongation (maximum excursion) was significantly higher.

Finally, the line-scanning spectrometer-based configuration may provide a balance between speeds and flexibility. Pandiyan et al.^71^ demonstrated an adaptive optics, line-scanning, spectrometer-based (AO-LS-SD) OCT capable of acquiring B-scans at 16 kHz, with an effective A-scan rate of 8 MHz. By varying scanning parameters, they were able to achieve volume rates of 324 Hz and 120 Hz, with which they characterized the initial contraction and later elongation of the cone OS. They showed a dose-dependence of both components and, using a slower volume rate of 15 Hz, showed that the amplitude of elongation responses to a red stimulus flash formed a trimodal distribution, hypothesizing that this was due to the trichromatic cone mosaic. This confirmed the findings of Zhang et al.^45^

Soon after, the same group improved their setup and used the highest volume rate (324 Hz) to study the kinetics and saturation of the early contraction component.^46^ Their study revealed that the onset of the contraction component occurred less than 1 ms after the stimulus onset. That observation, in conjunction with a detailed model of the electrostatic effects of photoisomerization on the cone opsins, led them to hypothesize that the mechanism of the early contraction component was the early receptor potential (ERP).^93^ In short, repulsion of activated opsins causes OS discs to expand laterally, which, in combination with conservation of disc surface area and OS volume, requires a contraction of the OS. Later, Pandiyan et al.^91^ showed that the elongation stage of the cone response was predicted best by a model consisting of two inverted exponential decay components with independent amplitudes and time constants. Based on the distinct kinetics of these two components, they hypothesized that the elongation of the OS is caused by (1) osmotic pressure due to increased phosphate concentration after PDE activation and (2) movement of water into the discs at high bleaching levels. The presence of two components was later confirmed by later AO-FF-SS-OCT work that showed two components fit elongation responses better than one, especially when bleaching levels were high (32%).^88^

## Optoretinography as a Marker of Dysfunction in Retinal Degenerations

The ultimate hope for ORG technologies is that they will be used to detect and quantify disease-related dysfunction. To date, there have been several preliminary investigations in this area.

Lassoued et al.^94^ used a MHz rate-scanning AO-SD-OCT system to measure phase-based cone responses in three patients with various stages of retinitis pigmentosa (RP). As with Zhang et al.,^45^ their analytical approach entailed concatenating cone responses to three colored stimuli (red, green, and blue) and performing principal component analysis on the concatenated responses. In the 2D space of the first two principal components, cones of the three spectral classes formed three distinct clusters. They defined a figure of merit, “relative response strength,” as the ratio of the distance of these clusters to those of a healthy volunteer. They showed that in cones with intact ISOS and COST reflections, the relative response strength declined with proximity to the RP lesion and also declined with disease progression.

Wendel et al.^95^ used an AO-LS-SD-OCT system to measure phase-based cone ORG responses in four RP patients, using an AO-SLO to measure cone densities and an AO-microperimetry system to study psychophysical detection sensitivity in parallel. Their metric for ORG response was the maximum elongation of the cone OS. They found that the ORG responses of RP patients exhibited reduced response and greater response variability than those of control subjects. They found some correlation between OS length and response, indicating that one of the factors in the reduced ORG response in patients is the overall shortening of outer segments due to disease. Finally, they noted some clustering of low-responding cones in two of the patients. In Liu et al.,^96^ the same group published longitudinal measurements in RP patients using the LS-SD-OCT system without AO. They imaged five patients, with two follow-up visits each, between 6 and 20 months after their initial visit. In all five patients, they observed decline in ORG amplitude and compared these declines with changes in the continuity of ISOS and changes in the length of the OS. They found that the loss of ORG amplitude across visits was more significant than changes in either of the structural biomarkers.

Xu et al.^97^ used a coherent illumination AO-SLO approach similar to Cooper et al.^79^ to show that choroideremia causes a reduction in ORG response. Their metric for response was essentially the standard deviation of reflectance among stimulated cones normalized by the standard deviation of reflectance among unstimulated cones. They found a reduction in this metric in patient measurements. Within patients, they observed correlations between ORG response and ELM-RPE distance (measured with commercial OCT) and cone density (measured in the structural AO-SLO images). Gaffney et al.^98^ applied a very similar AO-SLO–based method to show widespread reductions in ORG amplitude in seven patients with RP. Their study showed that the ORG was more sensitive to functional changes than microperimetry and also revealed changes in the morphology of photoreceptors.

Wongchaisuwat et al.^99^ reported SSADOR functional measurements in nine patients diagnosed with inherited retinal dystrophies, including three with RP. OCT SSADOR imaging was performed using a prototype ultra-high-resolution SD-OCT (1.8-µm axial resolution in the retina and 250-kHz A-scan rate). SSADOR consistently detected reductions in decorrelation that were spatially correlated with clinical lesions shown in fundus autofluorescence and visual field. SSADOR decorrelation also showed strong positive correlation to retinal sensitivity, measured using a microperimeter (MAIA Macular Integrity Assessment System). The study suggested that SSADOR may be more sensitive at detecting subtle cone function impairment at early disease stages than current clinical visual tests.

## Conclusion and the Near Future of the ORG

ORG promises an accessible, objective, and quantifiable approach to assess photoreceptor light responses. However, it has yet to achieve broad acceptance and definitive clinical indications. In this section, we describe a few research areas that could be especially important for fostering clinical translation of ORG methods.

### Standards for Stimulus Characterization

ORG methods, by definition, require delivery of visible stimuli to the retina. To synthesize the results of studies conducted by different groups, it is critical to have a standard language of stimuli. Our community is fortunate to be the intellectual heirs of the psychophysics, densitometry, and electroretinography communities, because methods for characterizing stimuli were standardized by them decades ago. However, there remain some areas of confusion. Prior to the development of the ORG, investigators in the area of imaging focused much more on structure than function. Imaging scientists tend to favor radiometric metrics while those interested in studying the sensation of vision favor photometric ones. Somewhere in between lies what might be the quantity most pertinent to studying the activation of photoreceptors—the number of photoisomerized opsins. Rushton and Henry^100^ provided a framework for calculating this number, but it depended on constants derived from experimental data on a few subjects. Vienola et al.^47^ provide a clearer recipe for calculating bleaching fractions from radiometric measurements (i.e., power and wavelength of the stimulus measured at the cornea), but these make the same assumptions made by Rushton and Henry.^100^ Zhang et al.^101^ offered an alternative method calculating photopigment bleaching, based on radiometric and other physical parameters (e.g., transmission of the eye, fill factor of the photoreceptors, cross-sectional area of the outer segment, packing density of opsins on the disc membrane, etc.), and the latter approach has been used in the ORG literature as well.^46^ Unfortunately, these two methods lead to estimates of photopigment bleaching that may differ by 20% or more, which hinders comparison of the respective cone responses.

One way out of the quandary would be for ORG investigators to comprehensively report the parameters related to photopigment bleaching. Ultimately, what is needed for reproduction and/or fair comparison of results is the stimulus energy incident on the retina, its spectrum, and its duration. From these quantities, researchers can use known spectral sensitivities of photoreceptor classes to derive the quantal catch and numbers of photoisomerizations. Since stimulus energy incident on the retina is not measured directly, the method by which it was derived should also be stated. Some of the papers reviewed here conform to this standard, for example, reports by Cooper et al.,^79^^,^^80^ Zhang et al.,^45^ Pandiyan et al.,^46^^,^^91^ Vienola et al.,^47^ Tomczewski et al.,^77^^,^^78^^,^^92^ Valente et al.,^88^ and subsequent publications from the same groups. Vienola et al.^47^ are noteworthy for the explicit statement of all equations and constants used to calculate bleaching fraction from power incident on the cornea.

### Standards for Quantification, or “Figures of Merit”

Once the stimulus has been characterized, the problem of quantifying the photoreceptor responses arises. To date, ad hoc figures of merit have generally been employed, often contingent on the imaging modality: maximum elongation, minimum contraction, maximum and minimum elongation/contraction velocity, pooled variance, decorrelation, and principal components of elongation. To the extent that AO-OCT methods have elucidated the physical changes in the OS in the greatest detail, these should be used to predict corresponding values of less physical metrics (pooled variance, decorrelation, and principal components), and these predictions should be tested experimentally. Results of such work would provide methods for translating the figures of merit among modalities.

### Predictive and Explanatory Modeling of ORG Responses

Modeling of the stimulus-evoked deformation of the OS is valuable for several key reasons. It has already been employed to propose explanations of the biochemical and biophysical origins of the effect,^46^^,^^91^^,^^92^ and these hypotheses could be tested using animal models or natural experiments offered by well-characterized genetic disorders in humans. Even in the absence of attendant mechanistic explanations, purely predictive models may have value as well.^88^ They permit parameterization of the ORG response, which provides multiple, potentially independent biomarkers of cone health and disease. Moreover, predictive models may provide analytical tools useful for interoperability and standardization of figures of merit.

### Characterization of ORG Sensitivity and Specificity

The potential for ORG to be adopted as a clinical tool depends on ways it can be shown to outperform existing functional assays and ways it can be shown to complement them (i.e., fill unmet needs). The dynamic range, sensitivity, and spatial resolution of existing functional assays such as perimetry and electroretinography (ERG) are well known. To know whether a proposed ORG modality outperforms these methods, comparisons must be made to its corresponding parameters. We should make efforts to do so, while highlighting any additional advantages of the ORG, such as its natural capacity to do simultaneous assessments of structure and function.

### Interoperability of ORG Methods

At present, compliance of ophthalmic imaging devices such as OCT and OCTA with interoperability standards such as DICOM is low.^102^ The causes for this low compliance are complex, but the ORG community should anticipate this problem and employ strategies to avoid it now, before such an effort is stymied by commercialization. Areas of investigation mentioned above, including stimulus standards, quantification, and predictive modeling, could be marshalled to support harmonization. Publication of exemplary raw data and data-processing pipelines would aid this effort as well.

### Development of Technologies

The “advanced” methods described above currently face obstacles to clinical adoption, mostly associated with the profitability of their commercial development. Higher-speed lasers, cameras, and computers add to the costs of instrument development. The most exotic methods, such as full-field methods and methods using AO, may impose additional costs in hardware, optical redesign, and data processing. There are a few ways the research community may help to foster commercialization and clinical adoption. If we can identify and demonstrate the economic benefits of ORG (e.g., in the duration of patient exams or efficiency of clinical trials), the additional cost of the equipment may be balanced by reductions of other costs. Alternatively, if critical technologies (e.g., faster light sources) are shown to be cost-effective for another purpose (e.g., OCT angiography), the ORG might piggyback on those developments.

The scientific value of cellular-resolution methods (including AO-SD-OCT, AO-LS-SD-OCT, AO-SS-OCT, and FF-SS-OCT with DAC) is not disputable. These methods were the first ones used to demonstrate phase-based functional morphometry,^43^ elucidate the stimulus-evoked signal in cones^61^ and rods,^20^ perform spectral classification,^45^ test biophysical hypotheses,^46^ and measure disease-related dysfunction.^94^ However, the method has limitations—high instrument cost, time- and personnel-intensive imaging procedures, large data volumes, and limited (∼1°) FOV. Its translational success will depend critically on technical developments. Natural decline in the cost of specialized optical and computer hardware may eventually make it affordable. The limited FOV of AO systems is due in part to the sampling density required to make use of its 2 to 3-µm resolution and in part to the isoplanatic patch—the spatial region over which aberrations can be corrected. Developments in multiconjugate AO^31^ could facilitate expansion of the isoplanatic patch, making possible AO images with wider, more clinically friendly, fields of view.

A final development in parallel detection methods (e.g., AO fundus imaging and FF-SS-OCT) is the use of methods derived from spinning disk microscopy to allow confocal rejection of multiply scattered or out-of-plane light in conjunction with areal illumination. Investigators in this area have used digital micromirror devices or rolling shutters to create patterns in illumination and/or detection, effectively creating arrays of confocal pinholes in the system.^103^^–^^106^ This approach has the potential to marry the high speeds achievable with AO fundus imaging (which can exceed 1 kHz with modern scientific CMOS cameras) with the confocality of AO-SLO imaging. Combined with coherent illumination, this approach could produce a high-speed, common-path interferometric ORG.
